# Knockout of Two Cry-Binding Aminopeptidase N Isoforms Does Not Change Susceptibility of *Aedes aegypti* Larvae to *Bacillus thuringiensis* subsp. *israelensis* Cry4Ba and Cry11Aa Toxins

**DOI:** 10.3390/insects12030223

**Published:** 2021-03-05

**Authors:** Junxiang Wang, Xiaozhen Yang, Huan He, Jingru Chen, Yuanyuan Liu, Wanting Huang, Luru Ou, Zhaohui Yang, Xiong Guan, Lingling Zhang, Songqing Wu

**Affiliations:** 1 State Key Laboratory of Ecological Pest Control for Fujian and Taiwan Crops, College of Plant Protection, Fujian Agriculture and Forestry University (FAFU), Fuzhou 350002, China; junxiangwang1201@163.com (J.W.); yangxiaozhen0907@163.com (X.Y.); hehuanpike@163.com (H.H.); cjr35057788@163.com (J.C.); liuyuanyuan2021@163.com (Y.L.); hwthuangwanting@163.com (W.H.); olr13395087102@163.com (L.O.); buibuiyang@163.com (Z.Y.); guanxfafu@126.com (X.G.); 2 College of Forestry, Fujian Agriculture and Forestry University (FAFU), Fuzhou 350002, China; 3 College of Life Science, Fujian Agriculture and Forestry University (FAFU), Fuzhou 350002, China; 4 Postdoctoral Station of Forestry, College of Forestry, Fujian Agriculture and Forestry University (FAFU), Fuzhou 350002, China

**Keywords:** *Aedes aegypti*, Cry4Ba, Cry11Aa, aminopeptidase N, CRISPR/Cas9

## Abstract

**Simple Summary:**

The midgut aminopeptidase N (APN) isoforms have been identified as the binding receptor of insecticidal Cry toxins in numerous insects, including the major arbovirus vector *Aedes aegypti* (*Ae. aegypti*). However, whether the Cry-binding APN acts as an essential functional receptor to mediate *Bacillus thuringiensis* subsp. *israelensis* (Bti) toxicity in *Ae. aegypti* larvae remains to be determined. In this study, our results provide the direct molecular evidence demonstrating that two Cry-binding APN isoforms (*Ae*APN1 and *Ae*APN2) did not play a key role in mediating Bti Cry4Ba and Cry11Aa toxicity in *Ae. aegypti* larvae.

**Abstract:**

The insecticidal Cry4Ba and Cry11Aa crystal proteins from *Bacillus thuringiensis* subsp. *israelensis* (Bti) are highly toxic to *Ae. aegypti* larvae. The glycosylphosphatidylinositol (GPI)-anchored APN was identified as an important membrane-bound receptor for multiple Cry toxins in numerous Lepidoptera, Coleoptera, and Diptera insects. However, there is no direct molecular evidence to link APN of *Ae. aegypti* to Bti toxicity *in vivo*. In this study, two Cry4Ba/Cry11Aa-binding *Ae. aegypti* GPI-APN isoforms (*Ae*APN1 and *Ae*APN2) were individually knocked-out using CRISPR/Cas9 mutagenesis, and the *Ae*APN1/*Ae*APN2 double-mutant homozygous strain was generated using the reverse genetics approach. ELISA assays showed that the high binding affinity of Cry4Ba and Cry11Aa protoxins to the midgut brush border membrane vesicles (BBMVs) from these APN knockouts was similar to the background from the wild-type (WT) strain. Likewise, the bioassay results showed that neither the single knockout of *Ae*APN1 or *Ae*APN2, nor the simultaneous disruption of *Ae*APN1 and *Ae*APN2 resulted in significant changes in susceptibility of *Ae. aegypti* larvae to Cry4Ba and Cry11Aa toxins. Accordingly, our results suggest that *Ae*APN1 and *Ae*APN2 may not mediate Bti Cry4Ba and Cry11Aa toxicity in *Ae. aegypti* larvae as their binding proteins.

## 1. Introduction

*Aedes aegypti* (*Ae. aegypti)* is a principal vector of several human diseases, including dengue fever, yellow fever, chikungunya, and Zika fever [1]. The annual dengue incidence was estimated to be 390 million infections worldwide by the World Health Organization (WHO) [2]. Unfortunately, no effective vaccines against some vector-borne viral diseases (i.e., chikungunya and Zika) have been developed to date [3,4]. Hence, there is an urgent need to establish an effective and comprehensive control system for reducing the population density of vector mosquitoes.

The microbial agents *Bacillus thuringiensis* (Bt) have been used worldwide for pest control in agriculture, forestry, and sanitation [5]. Bt subsp. *israelensis* (Bti) is one of the Bt subspecies that specifically kills mosquito larvae by secreting a variety of crystalline toxins (Cry4A, Cry4B, Cry10A, and Cry11A) and cytolytic toxins (Cyt1 and Cyt2) [6]. Bti has entered the WHO-recommended insecticide list against pathogenic mosquitoes due to its environmental safety profile, target specificity, and lack of potential for resistance development [7,8]. Nonetheless, the insecticidal mechanism of Bt has not been fully understood and remains under investigation [9,10]. According to the known model of action of the three-domain Cry (3d-Cry) toxins, the Cry protoxins are first hydrolyzed at the N-terminus by gut proteases of target insects, and then bind to multiple receptors in the midgut brush border. The activated Cry toxins further accumulate in the apical membrane of the epithelial cells to oligomerize, induce pore formation, and destroy the cell osmotic balance; mechanisms which eventually lead to midgut ulceration and larval death [10]. Currently, four major midgut molecules have been identified as receptors for lepidoptera-specific Cry1 toxins, including the cadherin-like (CAD), the glycosylphosphatidylinositol (GPI)-anchored aminopeptidase N (APN), the GPI-anchored alkaline phosphatase (ALP), and the ATP-binding cassette transporter subfamily C (ABCC2/3) [11]. Several recent studies have revealed that Cry resistance mechanisms were primarily associated with the genetic alterations of these membrane receptors in several field- and laboratory-selected Bt-resistant insects [12]. Hence, functional analysis of Bt receptors is the key to an in-depth understanding of the underlying molecular basis of Bt toxicity and resistance mechanisms in target insects.

The APN family (EC 3.4.11.2) is a ubiquitous hydrolase enzyme mainly responsible for digesting protein through hydrolyzing neutral and basic amino acids at the N-terminus of polypeptides [13]. Since a 120-kDa APN of *Manduca sexta* (*M. sexta*) was first identified as the Cry-binding receptor, many more Lepidoptera GPI-APNs have been proposed to be involved in the process of Bt toxin binding [14,15]. Similarly, APNs of *Anopheles quadrimaculatus* and *Anopheles gambiae* were identified as the binding receptors for the Bt subsp. *Jegathesan* (Btj) Cry11Ba toxin [16,17]. Two *Ae. aegypti* GPI-APN isoforms, named *Ae*APN1 (AAEL012778), *Ae*APN2 (AAEL008155, the VectorBase ID has been updated to AAEL019828) have been identified as Cry11Aa-binding receptors by pull-down assays and receptor-binding studies [18,19]. The toxicity of Cry11Aa against *Ae. aegypti* larvae was enhanced by adding the full-length and partial fragments of *Ae*APN2 proteins [19]. Thus, the above evidence suggested that *Ae. aegypti* GPI-APNs may be involved in the pathogenesis of different Bti Cry toxins as important membrane-bound receptors.

To investigate whether the Cry-binding APN is the essential functional receptor for Bti Cry toxins, CRISPR/Cas9-mediated mutagenesis was used for the functional analysis of *Ae*APN1 and *Ae*APN2 isoforms by loss-of-function approach in *Ae. aegypti*. Two *Ae*APNs are hypothesized to be the functional receptor molecules involved in the toxicity of Bti Cry4Ba and Cry11Aa, which in APN knockouts should be more tolerant to Cry4B and Cry11A toxins than the WT strain. However, our study showed that both the binding affinity and the larval susceptibility of the single *Ae*APN1 or *Ae*APN2 knockout strain, and the double *Ae*APN1/*Ae*APN2 mutant strain to Cry4B and Cry11A toxins did not differ significantly compared to the wild-type (WT) strain. Our results provide direct molecular evidence demonstrating that *Ae*APN1 and *Ae*APN2 may not mediate Bti Cry4Ba and Cry11Aa toxicity in *Ae. aegypti.*

## 2. Materials and Methods

### 2.1. Mosquito and Bt Strains

The laboratory WT strain of *Ae. aegypti* (*Ae. aegypti* Haikou strain) was provided by the Fujian International Travel Health Care Center (Fujian, Fuzhou, China) and reared without exposure to Bt toxins for over 50 generations. All *Ae. aegypti* strains were maintained at 26 ± 1 °C and 83% ± 3% relative humidity with a photoperiod of 14-h light/10-h dark cycles. The recombinant Bt strains individually producing Cry4Ba and Cry11Aa (pCG6-Cry4Ba and pCG6-Cry11Aa) were provided by Dr. Sarjeet R Gill’s Laboratory, University of California Riverside, USA and stored at −80 °C.

### 2.2. Purification of Cry4Ba and Cry11Aa Protoxins

The pCG6-Cry4Ba and pCG6-Cry11Aa were grown in the 1/2 LB (Luria-Bertani) medium containing 12.5 μg/mL erythromycin at 30 °C. Subsequently, the crystal inclusions were completely released from the spores, the medium was removed by centrifugation at 9500× *g* and the pellet were washed three times with 1 M NaCl plus 0.03% Triton X-100 and three times with distilled water. The Cry4Ba crystal inclusion was purified by a repeated crystal solubilization method as previously described [20]. The Cry11Aa crystal inclusion was purified by discontinuous sucrose gradients as previously described [21]. The Cry4Ba protoxin was solubilized in alkaline buffer (50 mM Na_2_CO_3_/NaHCO_3_, pH 10.5), and Cry11Aa protoxin was solubilized in distilled water. The protein concentrations of Cry protoxins were determined using the Bradford Protein Assay Kit (Beyotime, Shanghai, China) with Bovine Serum Albumin (BSA) as the standard.

### 2.3. sgRNA Design and Synthesis

The CRISPR/Cas9 target sites were designed in the third exon of *Ae*APN1 and *Ae*APN2 genes using CRISPOR program (http://crispor.tefor.net/ (accessed on 8 October 2019)), and potential off-target effects were evaluated by Cas-OFFinder (http://www.rgenome.net/cas-offinder/ (accessed on 8 October 2019)) (Table S1). DNA templates for small-guide RNAs (sgRNAs) were prepared by polymerase chain reaction (PCR) with target specific primers (Table 1) using KOD Hot Start Polymerase (TOYOBO, Osaka, Japan), and purified using a Gel Extraction Kit (OMEGA Bio-Tek, Norcross, GA, USA). In vitro transcription of sgRNAs was performed using the MEGAscript Kit (Ambion, Austin, MA, USA), and purified by phenol: chloroform extraction and isopropanol precipitation as per the manufacturer’s instructions. The purified sgRNA concentration was determined using the ultra-micro spectrophotometer Q5000 (Quawell, San Jose, CA, USA) and stored at −80 °C until use.

### 2.4. Embryo Microinjection and Generation of Ae. aegypti Knockout Strains

The collection of mosquito embryos and microinjections were performed as described previously [22]. About 1 nL sgRNA/Cas9 mixtures of 300 ng/μL *sp*Cas9 protein (NEB, USA), and 100 ng/μL each of sgRNAs and 1x Cas9 nuclease reaction buffer was injected into the fresh embryos using the Nanoject III™ Microinjection System (Drummond, PA, USA). The hatched G_0_ embryos were reared to pupation, and each pupa was transferred individually in cups for eclosion. The genomic DNA (gDNA) was extracted from one hindleg of each G_0_ mosquito adult using 50 μL of chelex-100 buffer containing 5% chelex-100 beads (BioRad, Berkeley, CA, USA) and 1 mg/μL Proteinase K (TaKaRa, Dalian, China), hatching at 55 °C overnight and 5 μL supernatant for PCR template. G_0_ mutations were identified by PCR amplification using Premix Taq Version 2.0 (TaKaRa, Dalian, China) with corresponding target specific primers (Table 1) followed by Sanger sequencing. The validated G_0_ mutations were single outcrossed with the WT strain to generate F_1_ families, and the F_1_ individuals were validated by PCR and sequencing as described above. F_1_ individuals with similar mutation sequences were pooled to generate F_2_ families. The homozygous mutants were screened from F_2_ individuals and pooled to establish the homozygous knockout strains.

### 2.5. Preparation of Brush Border Membrane Vesicles from Ae. aegypti Larvae

The alimentary tract tissues were dissected from approximately 500 early four-instar mosquito larvae, soaked in MET buffer (0.3 M mannitol, 5 mM EDTA, and 17 mM Tris-Cl, pH 7.5) and stored at −80 °C. The brush border membrane vesicles (BBMVs) were prepared using the differential magnesium precipitation method, as described previously [23]. The protein concentrations of the BBMVs were determined as described above.

### 2.6. Proteomic Identification of Midgut BBMVs from Ae. aegypti Larvae

To confirm the absence of *Ae*APN1 and *Ae*APN1 isoforms at the protein level in midgut brush border membrane of *Ae*APN knockouts, proteomic analysis of midgut BBMV proteins were performed by liquid chromatography-tandem mass spectrometry (LC-MS/MS). Then, 15 μg BBMV proteins were separated on 10% SDS-PAGE, and the target regions (~70 kDa−170 kDa) of Coomassie blue stained gels including *Ae*APNs were excised and stored at −20 °C. The gel pieces were in-gel digestion and LC-MS/MS analysis using an Orbitrap-Fusion-Tribrid mass spectrometer (Thermo Fisher Scientific, MA, USA) at the Basic Forestry and Proteomics Research Center, FAFU. The resulting MS/MS data were processed using Proteome Discoverer 1.3 were matched against the annotated protein databases of *Ae. aegypti* (https://vectorbase.org/vectorbase/app/downloads/Current_Release/AaegyptiLVP_AGWG/fasta/data/ (accessed on 1 February 2021)).

### 2.7. ELISA Binding Assays

ELISA binding assay was performed as reported previously [24]. The ELISA plates were coated with 0.3 μg *Ae. aegypti* BBMVs in a final volume of 100 uL PBS/well overnight at 4 °C. Then, the plates were washed three times with 200 μL PBS, and blocked with 200 μL/well of blocking buffer (PBS with 2% low fat milk powder) for 2 h at 37 °C. The plates were washed three times with 200 μL PBST and 100 μL/well of different concentrations of Cry protoxin/PBST were added and the cultures were incubated at 37 °C for 1 h. After washing three times with PBST and PBS to remove unbound protoxins, the wells were incubated with 100 μL blocking buffer containing anti-Cry4Ba or anti-Cry11Aa polyclonal antibody (1:10,000 dilution) for 1 h at 37 °C. After the required washing steps, 100 μL/well of PBST containing HRP-labeled goat anti-rabbit IgG (H + L) antibodies (1:5000 dilution) (Beyotime, Shanghai, China) was added and allowed to incubate for 1 h at 37 °C. The wells were washed again and 100 μL/well of TMB chromogenic solution (Beyotime, Shanghai, China) was added and allowed to react for 8–15 min at 37 °C, protected from light. Finally, 50 μL/well of 2 M H_2_SO_4_ was added to terminate the reaction, and the optical density (OD) value of each well was read at 450 nm with an absorbance microplate reader (BioTek, Winooski, VT, USA). The K*_d_* values were calculated using Curve Expert 1.4 (Hyams DG Softwave) and the ELISA-binding plots were generated in GraphPad prism 8.0 (GraphPad Softwave).

### 2.8. Bioassay of Bti Cry Toxins

The susceptibility to Bti Cry4Ba and Cry11Aa toxins was determined for different *Ae. aegypti* strains as described [25]. At least 5 gradient concentrations of purified Cry4Ba and Cry11Aa protoxins were added to 20 mL filtered water containing 25 early fourth instar larvae and mixed fully. Each larvae bioassay was repeated with three biological replications. The larval mortality was recorded after 24 h, and the medium lethal concentration (LC_50_) and the 95% confidence intervals (CI) of the LC_50_ were calculated by probit analysis using PoloPlus (LeOra Software).

## 3. Results

### 3.1. Generation of Individual AeAPN1 and AeAPN2 Knockout Ae. aegypti Strains by CRISPR/Cas9

**Figure 1:**
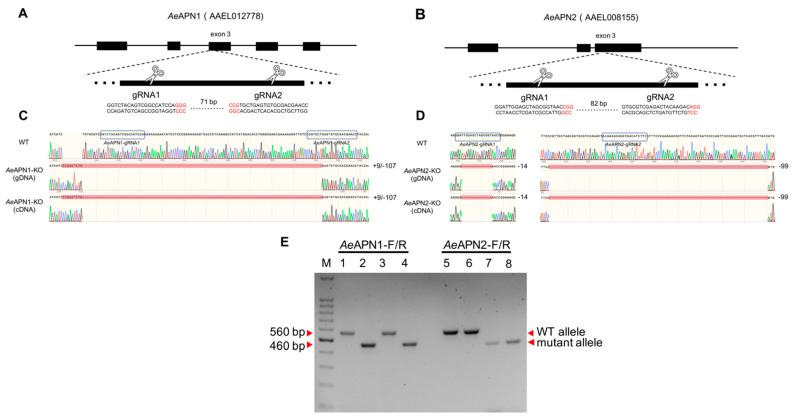
Generation of *Ae*APN knockout *Ae. aegypti* strains. Schematic representations of the *Ae*APN1 (**A**) and *Ae*APN2 (**B**) locus, where enlarged is the third exon contain 20-nucleotide sgRNA target sequences, and the PAM sequence shown in red. Aligned Sanger-sequencing trace of PCR-amplified using gDNA and cDNA from WT, *Ae*APN1-KO and *Ae*APN2-KO strains with specific primers (Table 1) spanning the gRNA targeted region (**C**,**D**). Electrophoresis of genomic-PCR products spanning target region from WT, *Ae*APN1-KO, *Ae*APN2-KO, and *Ae*APN1/*Ae*APN2-KO strains with specific primers *Ae*APN1-F/R and *Ae*APN2-F/R (**E**), Lane M: 100 bp DNA Ladder (TaKaRa, Dalian, China), Lane 1 and 5: the fragments were amplified from gDNA of the WT strain, Lane 2 and 6: the fragments were amplified from gDNA of the *Ae*APN1-KO strain, Lane 3 and 7: the fragments were amplified from gDNA of the *Ae*APN2-KO strain, Lane 4 and 8: the fragments were amplified from gDNA of the *Ae*APN1/*Ae*APN2-KO strain.

To increase G_0_ mutagenesis efficiency and to induce sequence fragment deletions for *Ae*APN1 and *Ae*APN2 gene regions, we designed two CRISPR/Cas9 target sites within 100 bp region of the corresponding third exons to disrupt the downstream GPI-anchored sites (Figure 1A, B). After 400 and 800 *Ae. aegypti* eggs were injected with sgRNA/Cas9 mixtures to knockout *Ae*APN1 and *Ae*APN2 genes, 26 and 88, respectively, they hatched into larvae within one month (Table 2). DNA sequencing of 560 bp gDNA fragments spanning the gRNA targeted region from *Ae*APN1- and *Ae*APN2-knockout G_0_ individuals indicated that 4 and 36 of the larvae, respectively, presented undefined peaks around the CRISPR/Cas9 target sites (Table 2). The homozygous knockout (KO) *Ae. aegypti* strain for *Ae*APN1 (named *Ae*APN1-KO) and APN2 (named *Ae*APN2-KO) were generated using the reverse genetics approach. The *Ae*APN1-KO presented a 107 bp deletion and 9 bp insertion between 2 of the CRISPR/Cas9 target sites, causing an amino acid mismatch and protein translation to terminate prematurely upstream of the GPI-anchored site (Figure 1C and Figure S1). The *Ae*APN2-KO had a 14 bp deletion and 99 bp deletion in the 2 of CRISPR/Cas9 target sites respectively, also resulting in the deletion of the GPI-anchored site and a loss of function as the membrane receptor (Figure 1D and Figure S2). The complementary DNA (cDNA) PCR sequencing of the two homozygous knockouts showed the genotypes of the coding region were consistent with the gDNA (Figure 1C,D).

### 3.2. Generation of AeAPN1/AeAPN2 Double-Mutant Ae. aegypti Strain

To generate a homozygous *Ae. aegypti* strain with double *Ae*APN1/*Ae*APN2 knockout, *Ae*APN1-KO males were crossed with *Ae*APN2-KO females, and the homozygous mutants (named *Ae*APN1/*Ae*APN2-KO) were screened by the reverse genetics approach described above. The PCR products of *Ae*APN1 and *Ae*APN2 mutant allele were significantly smaller than the WT allele, which were easily separated by gel electrophoresis (Figure 1E). The proteomic analysis of target midgut BBMV proteins (~70–170 kDa) from WT and *Ae*APN1/*Ae*APN2-KO strains by LC-MS/MS, showed that 1382 proteins were identified from WT sample including 43 unique peptides of *Ae*APN1 and 36 peptides (34 unique peptides) of *Ae*APN2, while no *Ae*APN1 and *Ae*APN2 peptides were identified in 1245 proteins of *Ae*APN1/*Ae*APN2-KO sample, suggesting the loss of GPI-anchoring signal of *Ae*APN1 and *Ae*APN2 cannot anchor to the midgut apical microvilli of *Ae. aegypti* (Figure S3 and Table S2).

### 3.3. Cry4Ba and Cry11Aa Protoxins Binding to BBMVs of the APN Knockouts and the WT Strains

**Figure 2:**
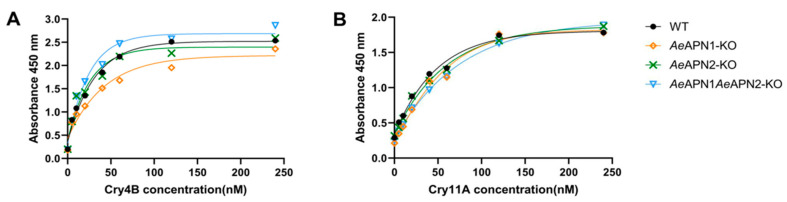
Binding of Cry4Ba and Cry11Aa toxins to *Ae.* *aegypti* BBMVs by ELISA. Different concentrations (0 nM, 5 nM, 10 nM, 20 nM, 40 nM, 60 nM, 120 nM, 240 nM) of Cry4Ba (**A**) and Cry11Aa (**B**) protoxins bound to 0.3 μg midgut BBMVs from APN mutant strains and WT strain in the ELISA plates.

The ELISA binding assays using the Cry4Ba protoxin to BBMVs of *Ae*APN1-KO, *Ae*APN2-KO, *Ae*APN1/*Ae*APN2-KO, and WT strains showed that the Cry4Ba bound to BBMVs of APN knockouts with high affinity (K*_d__Ae_*_APN1-KO_ = 7.82 nM, K*_d__Ae_*_APN2-KO_ = 8.31 nM and K*_d__Ae_*_APN1/*Ae*APN2-KO_ = 8.94 nM), which was not significantly different than the binding of the WT strain (K*_d_*_WT_ = 8.07 nM) (Figure 2A). The Cry11Aa protoxin also had a high affinity to BBMVs of APN knockouts (K*_d__Ae_*_APN1-KO_ = 9.9 nM, K*_d__Ae_*_APN2-KO_ = 11.01 nM, K*_d__Ae_*_APN1/*Ae*APN2-KO_ = 10.35 nM, and K*_d_*_WT_ = 7.88 nM) (Figure 2B). These data indicated that losing of *Ae*APN1 and *Ae*APN2 cannot affect the high affinity of Cry4Ba and Cry11Aa toxins binding the midgut epithelial cells of *Ae. aegypti* larvae.

### 3.4. Response of the Ae. aegypti Larvae to Cry4Ba and Cry11Aa Protoxins

Bioassays of the *Ae*APN1-KO, *Ae*APN2-KO, and *Ae*APN1/*Ae*APN2-KO strains with Cry4Ba and Cry11Aa showed that the LC_50_ of Cry4Ba and Cry11Aa for these three APN mutant strains were similar to that of the WT strain, which indicated that neither the single knockout of *Ae*APN1 or *Ae*APN2, nor simultaneous disruption of *Ae*APN1 and *Ae*APN2 resulted in a significant reduction of *Ae. aegypti* larvae susceptibility to Cry4Ba and Cry11Aa toxins (Table 3 and Table 4).

## 4. Discussion

In recent decades, the identification of midgut molecules as Bt receptors in target insects has been a research objective to define Bt activity. The sequential binding model proposes that the activated Cry toxin forms a Pre-pore oligomer after interacting with the transmembrane receptor CAD, and further binding to the GPI-anchored membrane receptors (APN or ALP) leads to membrane insertion, pore formation, and cell lysis [14]. The GPI-APN was the first to be described as a Cry-binding receptor in the midgut of Lepidopteran insects [15]. However, whether the Cry-binding APN acts as an essential functional receptor to mediate Bti toxicity in *Ae. aegypti* larvae remains to be determined. In this study, our results demonstrated that deficiency in *Ae*APN1 and *Ae*APN2 isoforms do not affect either the binding affinity or susceptibility of *Ae. aegypti* larvae to Cry4Ba and Cry11Aa toxins.

Early functional analyses of APN as Cry receptors showed that the GPI-APNs of *M. sexta* and *Heliothis virescens* (*H. virescens*) were reconstituted into phospholipid vesicles and planar lipid bilayers to increase the binding affinity of Cry1 toxins and catalyze channel formation [26,27,28]. The in vitro cytotoxicity analysis indicated that heterologous expression of different APNs in insect cell lines (S2, Sf9, and Sf21), which are not susceptible to Cry toxin, decrease the cell viability or facilitate cell swelling under activated Cry toxins infection, such as following *Helicoverpa armigera* (*H. armigera*) APN1 [29], *H. virescens* APN1 [30] and two of *Ae. aegypti* APNs [31] infections. Whereas, the expression of *M. sexta* APN1 in S2 cells did not lead to host cell sensitivity to activated Cry1 toxins [32]. Furthermore, the down-regulated APN expression using *in vivo* RNAi was associated with tolerance to Cry toxins in several insects, including *Spodoptera exigua* APN1 [33], *M. sexta* APN1 [34], three *Chilo suppressalis* APNs [35], three *Diatraea saccharalis* APNs [36] and three *Ae. aegypti* APNs [37]. Another important source of APN involvement in Bt toxicity is suggested by deletion mutation studies of *Ha*APN1 and the down-regulation of *Trichoplusia ni* (*T. ni*) APN1 transcription, which have been shown to be genetically linked with Cry1Ac resistance [38,39]. The transcriptome analysis of a laboratory-selected Cry11Aa resistant *Ae. aegypti* strain indicated that the transcript levels of two APNs (AAEL008158 and AAEL008162) were significantly down-regulated compared to the WT strain. Nonetheless, no changes in expression and non-synonymous mutations have been observed in *Ae*APN1 and *Ae*APN2 [40]. Thus, further functional analysis of APN applying genome-editing strategies are required to confirm the role of APN as a Bt functional receptor rather than as a Cry-binding protein.

Recently, CRISPR/Cas9-mediated genomic editing technology has provided a powerful tool to generate KO/knock-in models, and has been applied to the identification of Bt receptors in some lepidopteran insects. For example, the ABCA2 mutant generated by CRISPR/Cas9 in *T. ni* and *H. armigera* resulting in high-level resistance to Cry2A toxins [41,42]. CRISPR/Cas9-mediated double knockout of ABCC2 and ABCC3 in *H. armigera* and *Plutella xylostella* (*P. xylostella)* resulted in more than 1000-fold resistance to Cry1Ac [43,44]. KO of *T. ni* CAD and *Spodoptera frugiperda* (*S. frugiperda*) CAD did not affect the larvae susceptibility to Cry1 and Cry2 toxins [45,46]. Conversely, knockout of *H. armigera* CAD by CRISPR/Cas9 in a Bt-susceptible strain could increase resistance to Cry1Ac toxin by more than 500-fold [47]. Moreover, none of the 3 *H. armigera* APNs (*Ha*APN1, *Ha*APN2, and *Ha*APN5) individually knocked out using CRISPR/Cas9 resulted in any change in susceptibility of the larvae to Cry1A and Cry2A toxins [48].

Genetic and molecular studies have indicated that the entomopathogenicity of Bt is complex and may involve multiple membrane-bound receptors and intracellular pathways [9,49]. Generating single-receptor knockout insects by genome editing often does not achieve significantly different results in larvae susceptibility to Bt toxins. For example, neither *Px*ABCC2 nor *Px*ABCC3 knockout in *P. xylostella* strains produced any significant resistance to Cry1Ac, while simultaneous mutations of the two genes exhibited high-level resistance (>8000-fold) to Cry1Ac, revealing the functional redundancy between ABCC2 and ABCC3 as receptors in the activity of Cry1Ac toxins [44]. In this study, the double mutant *Ae*APN1/*Ae*APN2-KO strain did not show increased tolerance to Cry4Ba and Cry11Aa toxins, suggesting *Ae*APN1 and *Ae*APN2 did not exhibit synergistic effects on mediating the entomopathogenicity of Cry4Ba and Cry11Aa (Table 2 and Table 3). The genome-wide analysis of the APN gene family showed that 29 APN isoforms were identified in *Ae. aegypti* genome, 11 of them were predicted to carry the GPI-anchoring signal. Moreover, a previous pulldown assay and our Co-immunoprecipitation assay (unpublished data) showed that *Ae*APN3 (AAEL012774) could bind to Cry4Ba and Cry11Aa toxins in the midgut BBMVs of *Ae. aegypti* [18]. Therefore, the potential complementary roles of other *Ae*APNs as Cry-binding receptors in determining susceptibility to Cry4Ba/Cry11Aa toxins should not be ruled out. Comprehensive determination of the role of APN in the action mechanism of Bt requires further functional analysis of multiple APNs in a variety of insects. Overall, our study revealed that two Cry-binding APNs (*Ae*APN1 and *Ae*APN2) may not play a key role in mediating Bti Cry4Ba and Cry11Aa toxicity in *Ae. aegypti*.

## Figures and Tables

**Table 1:** List of DNA oligo primers used in this study.

Primer Name	Sequence 5′–3′
sgRNA-R	ATAACGGACTAGCCTTATTTTAACTTGCTATTTCTAGCTCTAAAAC
APN1-sgRNA-1-F	GAAATTAATACGACTCACTATAGGTCTACAGTCGGCCATCCAGTT TTAGAGCTAGAAATAGC
APN1-sgRNA-2-F	GAAATTAATACGACTCACTATAGGTTCGTCGCACACTCAGCAGTT TTAGAGCTAGAAATAGC
APN2-sgRNA-1-F	GAAATTAATACGACTCACTATAGGATTGGAGCTAGCGGTAACGTT TTAGAGCTAGAAATAGC
APN2-sgRNA-2-F	GAAATTAATACGACTCACTATAGTGCGTCGAGACTACAAGACGTT TTAGAGCTAGAAATAGC
*Ae*APN1-F	GGAATGCCGATACTCCAAGATCAAT
*Ae*APN1-R	TGAAAATAATCCACTCATTGGCCGG
*Ae*APN2-F	AGTGTTCTGAACATGTTCCGTGT
*Ae*APN2-R	TATGCGTCGTTGATCAGCTGAGC

**Table 2:** Transformation data of G_0_ embryos injected with CRISPR/Cas9 constructs.

Injected Component	Injected G_0_ Embryos	G_0_ Survivors	G_0_ Mutants
Cas9 protein, APN1-sgRNA-1 and APN1-sgRNA-2	400	26	4
Cas9 protein, APN2-sgRNA-1 and APN2-sgRNA-2	800	88	36

**Table 3:** Susceptibility of *Ae. aegypti* strains to Cry4Ba toxin.

*Ae. aegypti* Strain	n	Slope (SE)	LC_50_ (μg/mL) (95% CI)	RR ^a^
WT	1125	2.947 (0.098)	1.771 (1.663–1.888)	1
*Ae*APN1-KO	1125	3.774 (0.181)	1.504 (1.421–1.591)	0.849
*Ae*APN2-KO	1125	3.664 (0.171)	1.863 (1.771–1.958)	1.052
*Ae*APN1/*Ae*APN2-KO	1125	3.079 (0.135)	2.221 (2.092–2.367)	1.254

^a^ RR (Relative Resistance) = LC_50_ of the knockout strain/LC_50_ of the WT strain.

**Table 4:** Susceptibility of *Ae. aegypti* strains to Cry11Aa toxin.

*Ae. aegypti* Strain	n	Slope (SE)	LC_50_ (μg/mL) (95% CI)	RR ^a^
WT	1125	1.747 (0.110)	0.602 (0.526–0.685)	1
*Ae*APN1-KO	1125	3.192 (0.224)	0.556 (0.496–0.619)	0.924
*Ae*APN2-KO	1125	1.989 (0.124)	0.599 (0.546–0.653)	0.995
*Ae*APN1/*Ae*APN2-KO	1125	2.770 (0.163)	0.826 (0.769–0.897)	1.372

^a^ RR (Relative Resistance) = LC_50_ of the knockout strain/LC_50_ of the WT strain.

## Data Availability

Exclude this statement.

## Supplementary Materials

The following are available online at https://www.mdpi.com/2075-4450/12/3/223/s1, Figure S1: Amino acid sequence alignment of *Ae*APN1 isoform from WT and *Ae*APN1-KO strains, Figure S2: Amino acid sequence alignment of *Ae*APN2 isoform from WT and *Ae*APN1-KO strains, Figure S3: SDS-PAGE profile of midgut BBMV protein from the WT strain and the *Ae*APN1/*Ae*APN2-KO strain, Table S1: In silico analysis of off-target activity of sgRNAs used in this study, Table S2: *Ae*APN1 and *Ae*APN2 peptides identified from midgut BBMV of the WT strain.
