# Supplementary material for: Knockout of Two Cry-Binding Aminopeptidase N Isoforms Does Not Change Susceptibility of Aedes aegypti Larvae to Bacillus thuringiensis subsp. israelensis Cry4Ba and Cry11Aa Toxins
Source: Insects. 2021 Mar 5;12(3):223. doi: 10.3390/insects12030223 (PMC8002144; doi:10.3390/insects12030223)
Supplement: Supplementary file 1 [file insects-12-00223-s001.pdf]

Table S1. *In silico* analysis of off-target activity of sgRNAs used in this study

	Target	Chromosome	Position	Direction	Mismatches	Bulge Size
*Ae*APN1	**crRNA: GGATTGGAGCTAGCGGTAACNGG DNA: GGATTGGAGCTAGCGGTAACCGG**	3	9134967	+	0	0
	**crRNA: GTGCGTCGAGACTACAAGACNGG DNA: GTGCGTCGAGACTACAAGACAGG**	3	9135072	+	0	0
*Ae*APN2	**crRNA: GGTCTACAGTCGGCCATCCANGG DNA: GGTCTACAGTCGGCCATCCAGGG**	1	290625078	+	0	0
	**crRNA: GGTTCGTCGCACACTCAGCANGG DNA: GGTTCGTCGCACACTCAGCACGG**	1	290625172	-	0	0

```
             1         10        20        30        40        50
WT           MIVLKWSVVLWGLTLATYGTVAERPLSDVSDSVDPPQIAPAFAVEESEIIPLQEVDES
AeAPN1-KO    MIVLKWSVVLWGLTLATYGTVAERPLSDVSDSVDPPQIAPAFAVEESEIIPLQEVDES

             60        70        80        90        100       110
WT           YRLPKTSYPTHYELRLRTEVHTGNRQFDGTVAIHLNVVEATNAIVVHRRSLTIQKANL
AeAPN1-KO    YRLPKTSYPTHYELRLRTEVHTGNRQFDGTVAIHLNVVEATNAIVVHRRSLTIQKANL

             120       130       140       150       160       170
WT           AFIPTPEADPQQLNDPTWTYDANVEQLSFNSETLLNPGSYILTVEYNGRLSDSEDGFY
AeAPN1-KO    AFIPTPEADPQQLNDPTWTYDANVEQLSFNSETLLNPGSYILTVEYNGRLSDSEDGFY

             180       190       200       210       220       230
WT           ISSYVNKDGVTKYLATTQFESTSARMAFPCYDEPGLKATFALWITHDVLYTANSNMPY
AeAPN1-KO    ISSYVNKDGVTKYLATTQFESTSARMAFPCYDEPGLKATFALWITHDVLYTANSNMPY

             240       250       260       270       280       290
WT           TTTIDGDIRVTQFEVTPKMSTYLLAFVVSDFQRLGTLEHSVYARPNAIDEVVFAVEAG
AeAPN1-KO    TTTIDGDIRVTQFEVTPKMSTYLLAFVVSDFQRLGTLEHSVYARPNAIDEVVFAVEAG

             300       310       320       330       340
WT           QKILEKLDAHLGIGYYDHMPQMKQFAIPDFAAGAMENWGLVTYREQYLLFNPELSTYR
AeAPN1-KO    QKILEKLDAHLGIGYYDHMPQMKQFAIPDFAAGAMENWGLVTYREQYLLFNPELSTYR

             350       360       370       380       390       400
WT           TKTNIATVIAHEYAHQWFGNLVSPEWWEYIWLNEGFATLYEYYATHLAYPEVGYWELF
AeAPN1-KO    TKTNIATVIAHEYAHQWFGNLVSPEWWEYIWLNEGFATLYEYYATHLAYPEVGYWELF

             410       420       430       440       450       460
WT           NTQVIQAAMVPDGLETTRPMTWNADTPRSIASLFDRVAYPKSGSVLNMMRNVLGEINW
AeAPN1-KO    NTQVIQAAMVPDGLETTRPMTWNADTPRSIASLFDRVAYPKSGSVLNMMRNVLGEINW

             470       480       490       500       510       520
WT           TAGLKAYLTARQFDGANADHLYVGLQSAIQGKNVLPEGVTVKAIMDTWANEKGYPVLS
AeAPN1-KO    TAGLKAYLTARQFDGANADHPDSGVCDEPTKPEILSSRKSGSSRIVRFPIRTYG....

             530       540       550       560       570       580
WT           VRRTYETGDIIISQERFISDRKVPNTNVWMIPYNYVHQSKADFDDLSTFSWLSTKAAR
AeAPN1-KO    ..........................................................

             590       600       610       620       630
WT           INTEVPANEWIIFNKQQVGYYRVNYDANNWELITNALINNLSSIDRLNRAQLIDDAYW
AeAPN1-KO    ..........................................................

             640       650       660       670       680       690
WT           LARSGRLDIEVLMKLLTYLKDETEYAPWTAANNVLSYFNGKLRGTPAYKDFTTMVDHL
AeAPN1-KO    ..........................................................

             700       710       720       730       740       750
WT           IKKVYKTLDVTAVSDTEPLLHKYLKQTISTWACLIGNEDCLKRTKEALQKEVTEGIPV
AeAPN1-KO    ..........................................................

             760       770       780       790       800       810
WT           HPDVATVVYCNGLRTADVAEYQYLYKRIYPTQNWAFRSMIISALGCSENKQFLKDFLQ
AeAPN1-KO    ..........................................................

             820       830       840       850       860       870
WT           TAIGGSGSGVEINYKTAERTQIVQAVYSGGRAGVDALIEFLRDPNMLREFVAILDVNT
AeAPN1-KO    ..........................................................

             880       890       900       910       920
WT           LNSALSNIASRTNTQEELDMLNDLIVDLGTYITPQTAEAARATVQANFAWQQSAEAIL
AeAPN1-KO    ..........................................................

             930       940       950       960       970       980
WT           TMNFVGDFVDSIEPEPTTTVSPDSTTVSPASSTTAGSTTVAGSSTTEDDGGAATIAVS
AeAPN1-KO    ..........................................................

             990       1000
WT           VTLLIGAVAVALFN
AeAPN1-KO    ..............
```

Figure S1. Amino acid sequence alignment of AeAPN1 isoform from WT and AeAPN1-KO strains.

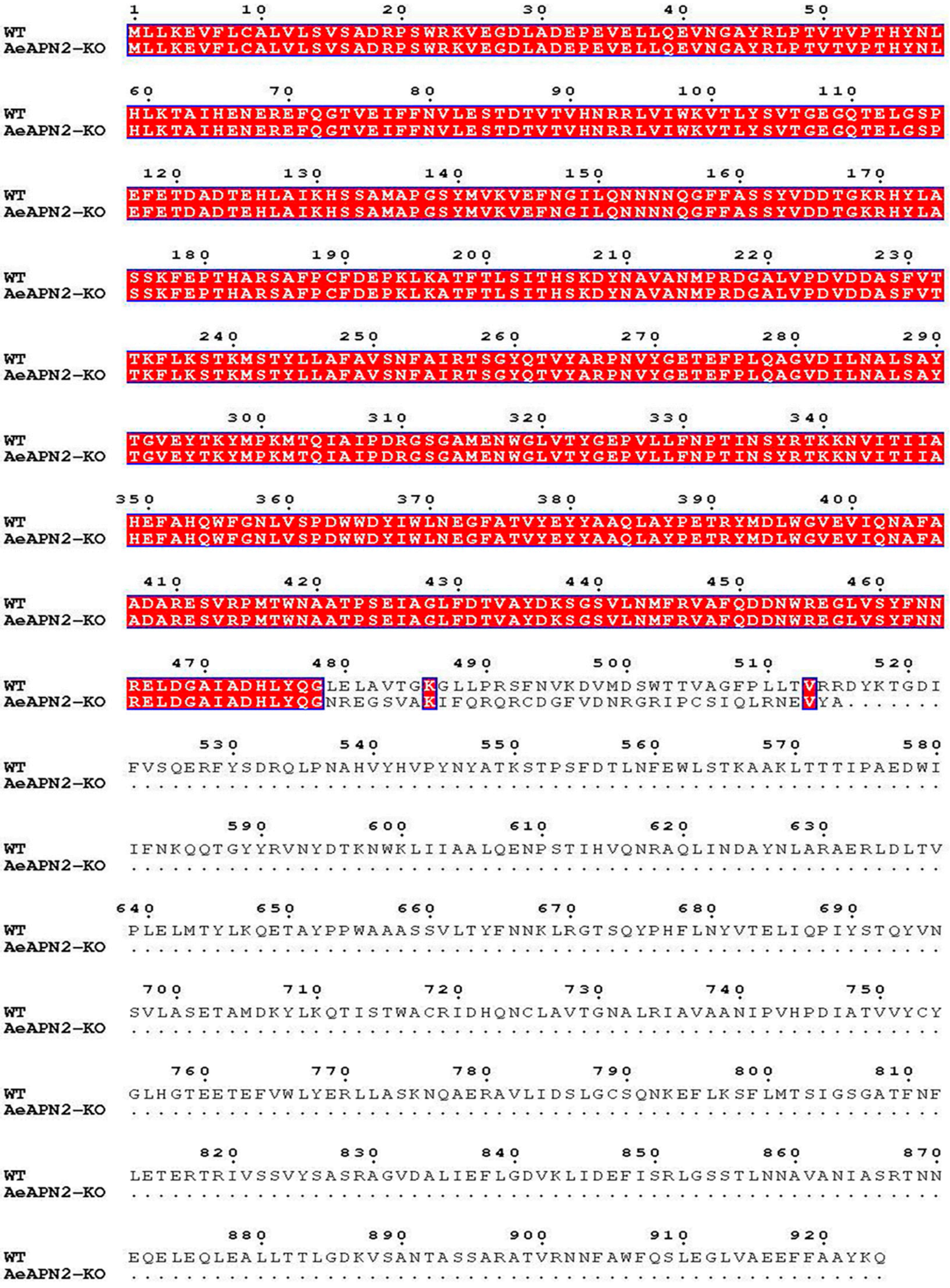

Figure S2. Amino acid sequence alignment of AeAPN2 isoform from WT and AeAPN1-KO strains.

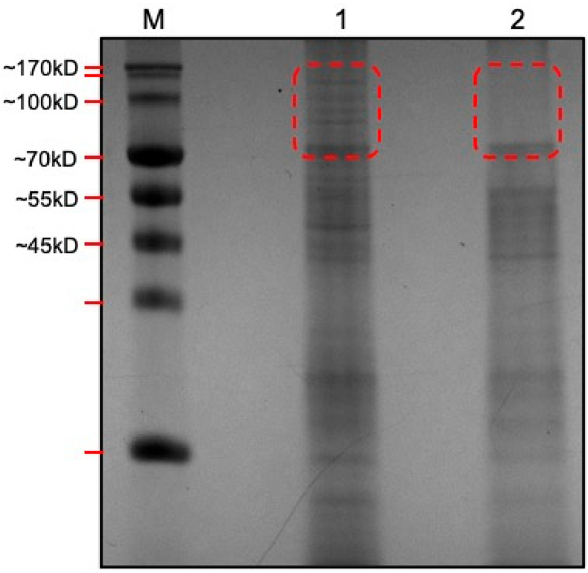

Figure S3. SDS-PAGE profile of midgut BBMV protein from the WT strain and the *Ae*APN1/*Ae*APN2-KO strain, and the target regions of Coomassie blue stained gels were framed by red dashed boxes. Lane M: Prestained Color Protein Ladder (Beyotime, China), Lane 1: the midgut BBMV proteins from the WT strain, Lane 2: the midgut BBMV proteins from the *Ae*APN1/*Ae*APN2-KO strain.

Table S2. *Ae*APN1 and *Ae*APN2 peptides identified from midgut BBMV of the WT strain

***Ae*APN1 peptides**		***Ae*APN2 peptides**	
IYPTQNWAFR	TLDVTAVSDTEPLLHK	MTQIAIPDR	EGLVSYFNNR
MAFPCYDEPGLK	DFLQTAIGGSGSGVEINYK	IVSSVYSASR	DVMDSWTTVAGFPLLTVR
QFAIPDFAAGAMENWGLVTYR	DFTTMVDHLIK	QLPNAHVYHVPYNYATK	DYNAVANMPR
QFDGANADHLYVGLQSAIQGK	DETEYAPWTAANNVLSYFNGK	LIIAALQENPSTIHVQNR	ESVRPMTWNAATPSEIAGLFDTVAYDK
RIYPTQNWAFR	EFVAILDVNTLNSALSNIASR	LGSSTLNNAVANIASR	IDHQNCLAVTGNALR
QTISTWACLIGNEDCLK	EQYLLFNPELSTYR	LIDEFISR	LDLTVPLELMTYLK
NVLGEINWTAGLK	AGVDALIEFLR	LTTTIPAEDWIIFNK	GSGAMENWGLVTYGEPVLLFNPTINSYR

NVLPEGVTVK	AGVDALIEFLRDP NMLR	LPTVTVPTHYNLHLK	HSSAMAPGSYMVK
INTEVPANEWIIF NK	KVPNTNVWMIPY NYVHQSK	VSANTASSAR	AQLINDAYNLAR
LSDSEDGFYISSYV NK	ADFDDLSTFSWLS TK	VTLYSVTGEGQTELGSPE FETDADTEHLAIK	MTQIAIPDR
VPNTNVWMIPYN YVHQSK	LDIEVLMK	VEFNGILQNNNNQGFFA SSYVDDTGKR	QETAYPPWAAASSVLT YFNNK
VTQFEVTPK	LDAHLGIGYYDH MPQMK	TAIHENER	FEPTHAR
YLATTQFESTSAR	AIMDTWANEK	STPSFDTLNFEWLSTK	QQTGYYR (AAEL018249-PB; AAEL019828-PA; AAEL012783-PA)
SMIISALGCSENK	GTPAYKDFTTMV DHLIK	SAFPCFDEPK	VNYDTK
TADVAEYQYLYK	AQLIDDAYWLAR	SGSVLNMFR (AAEL019828-PA; AAEL008163-PA)	
RTYETGDIIISQER	AGVDALIEFLRDP NMLR	VAFQDDNWR	
SIASLFDR	LDAHLGIGYYDH MPQMK	TNNEQELEQLEALLTTL GDK	
SGSVLNMMR	MSTYLLAFVVSDF QR	TGDIFVSQER	
TQIVQAVYSGGR	GYPVLSVR	DGALVPDVDDASFVTTK	
TYETGDIIISQER	QQVGYYR	ATFTLSITHSK	
TSYPTHYELR	LLTYLK	AVLIDSLGCSQNK	
DPNMLR		ELDGAIADHLYQGLELA VTGK	
